# Novel antimicrobial strategies to treat multi‐drug resistant *Staphylococcus aureus* infections

**DOI:** 10.1111/1751-7915.14268

**Published:** 2023-05-13

**Authors:** Edward J. A. Douglas, Sri W. Wulandari, Scott D. Lovell, Maisem Laabei

**Affiliations:** ^1^ Department of Life Sciences University of Bath Bath UK

## Abstract

Antimicrobial resistance is a major obstacle for the treatment of infectious diseases and currently represents one of the most significant threats to global health. *Staphylococcus aureus* remains a formidable human pathogen with high mortality rates associated with severe systemic infections. *S. aureus* has become notorious as a multidrug resistant bacterium, which when combined with its extensive arsenal of virulence factors that exacerbate disease, culminates in an incredibly challenging pathogen to treat clinically. Compounding this major health issue is the lack of antibiotic discovery and development, with only two new classes of antibiotics approved for clinical use in the last 20 years. Combined efforts from the scientific community have reacted to the threat of dwindling treatment options to combat *S. aureus* disease in several innovative and exciting developments. This review describes current and future antimicrobial strategies aimed at treating staphylococcal colonization and/or disease, examining therapies that show significant promise at the preclinical development stage to approaches that are currently being investigated in clinical trials.

## Graphical abstract

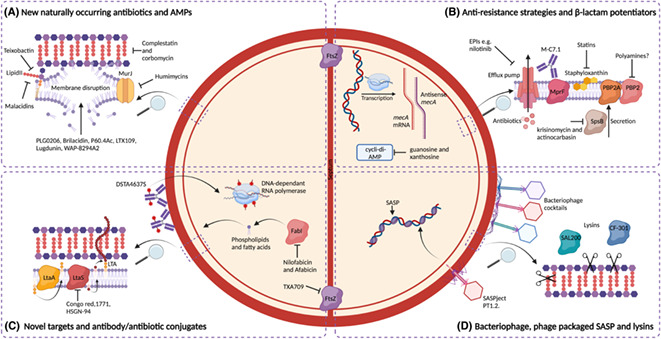

## INTRODUCTION

*Staphylococcus aureus* is a Gram‐positive, coagulase‐positive, non‐spore forming and non‐motile coccoid bacterium. It is widely regarded as one of the most impressive and successful human pathogens. These Janus‐faced bacteria (Broker et al., 2014) have evolved the capacity to inhabit the mammalian host asymptomatically while retaining the ability to transmit successfully in both healthcare and community environments and cause severe, often life‐threatening infections. *S. aureus* is well adapted to the human host and resides within the moist squamous epithelium of the anterior nares in 20–40% of the general population (Peacock et al., 2001). Following breach of cutaneous and/or mucosal barriers, *S. aureus* may gain access to the bloodstream or a underlying tissue, resulting in infection. Immunocompromised patients, persons with indwelling medical devices and people who are colonized by *S. aureus*, are at greatest risk to infection (Lowy, 1998; von Eiff et al., 2001).

*S. aureus* has evolved an extensive panel of multifunctional and often redundant secreted and cell wall anchored virulence factors which target components of both the innate and adaptive immune system and permit efficient adherence and colonization of specific anatomical niches (Cheung et al., 2021; Geoghegan & Foster, 2017). Consequently, *S. aureus* exists as a formidable opportunistic pathogen and a leading cause of skin and soft tissue infections, device‐associated infections, bacteraemia, endocarditis, bone and joint infections and pneumonia (Tong et al., 2015).

The introduction of antibiotics into medicine was arguably the most important medical development of the last century, however, shortly after the use of β‐lactam antibiotics in the clinic, antibiotic‐resistant *S. aureus* strains emerged (Kirby, 1944). Methicillin‐resistant *S. aureus* (MRSA), a term synonymous with resistance to an extensive list of β‐lactams, evolved following the acquisition of the mobile genetic element designated staphylococcal cassette chromosome *mec* (SCC*mec*) which harbours the *mecA* gene (Katayama et al., 2000). The gene *mecA* encodes the alternative penicillin‐binding protein 2a (PBP2a) which has a lower affinity for β‐lactams and can consequently perform essential crosslinking of the bacterial peptidoglycan in the presence of antibiotics (Hartman & Tomasz, 1984). Globally, MRSA spread rapidly in the healthcare and community settings via the serial emergence of dominant, geographically varied MRSA clones (Lee et al., 2018).

Although in recent years the incidences of healthcare‐associated MRSA infections have declined in China, Europe and the United States, they still present a significant clinical challenge in low‐ and middle‐income countries (Bai et al., 2022). Despite this decline, data from the United States indicate mortality due to MRSA infection remains higher than any other antibiotic resistant pathogen, with over 20,000 deaths reported in 2018 (Kourtis et al., 2019). In Europe, recent data show MRSA causes 150,000 infections and 7000 deaths annually (Cassini et al., 2019). In the most up‐to‐date study estimating global antimicrobial resistance (AMR) burden, MRSA caused more than 100,000 deaths in 2019 and *S. aureus* was listed as second in the top six pathogens for deaths associated with AMR (Antimicrobial Resistance Collaborators, Murray, 2022). Economically, MRSA infections are estimated to contribute to an additional $1.7 billion in extra in‐hospital costs for USA healthcare services every year (CDC, 2019).

At present MRSA is endemic in many healthcare facilities and global data confirm the major clinical challenge presented by multidrug resistant *S. aureus*. Currently there is a paucity in the development of novel classes of antibiotics required to combat antibiotic‐resistant infections. In addition, despite combined efforts from scientists and the pharmaceutical industry there is no licenced vaccine to prevent staphylococcal infection. Therefore, there is a major need to develop new antimicrobial strategies to tackle *S. aureus* disease.

In this review we examine potential antimicrobial strategies for the future treatment of staphylococcal disease, highlighting approaches that are in their infancy at preclinical development to treatments undergoing review in clinical trials. Not included for commentary are structural alternatives to existing antibiotic classes that are in various stages of clinical development which have been recently reviewed (Butler & Paterson, 2020; Hutchings et al., 2019).

## NOVEL TARGETS

### Lipoteichoic acid inhibitors

Teichoic acids are a family of diverse cell surface glycopolymers consisting of phosphodiester‐linked polyol repeat units. These polymers are divided into lipoteichoic acids (LTAs), which are linked to the membrane through a glycolipid anchor and wall teichoic acids (WTAs), polymers that are covalently attached to the peptidoglycan (Brown et al., 2013; Percy & Grundling, 2014). Both WTA and LTA play important roles in many aspects of cell physiology (Brown et al., 2013; Percy & Grundling, 2014). Importantly, mutants defective in WTA have growth and virulence impairments (Brown et al., 2013), whereas LTA mutants are conditionally lethal, requiring growth in high‐osmolarity medium and low temperature or rescued following the acquisition of compensatory mutations (Percy & Grundling, 2014). Accordingly, inhibition of teichoic acid biosynthesis is an attractive therapeutic intervention strategy, however, recent successes have centred on interrupting LTA biosynthesis.

LTA polymerization is initiated following the transfer of the diglucosyl‐diacylglycerol (Glc2‐DAG) lipid anchor from the cytoplasm to the membrane, likely via the action of the flippase LtaA. LTA synthesis requires the extension of the LTA glycerolphosphate (GroP) chain and linkage to the membrane anchor. This process involves the LTA synthase (LtaS) which promotes the repeated addition of GroP subunits sourced from the phosphatidylglycerol (PG) membrane lipids (Percy & Grundling, 2014). Several LTA inhibitors have been discovered and may prove to be effective future antibiotics.

**FIGURE 1:**
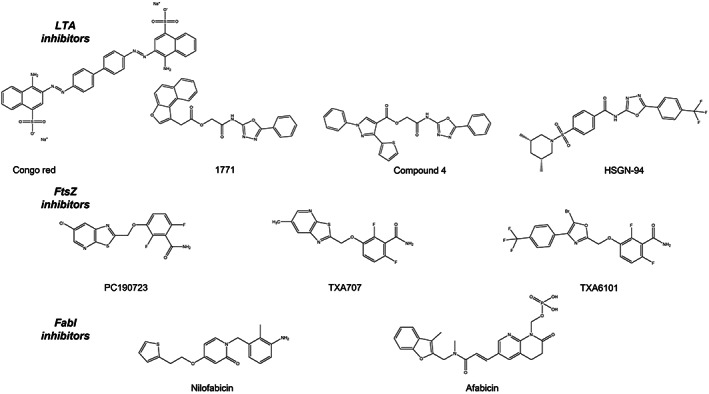
Structures of recently developed lipoteichoic acid (LTA), FtsZ and FabI inhibitors.

Compound 1771 [2‐oxo‐2‐(5‐phenyl‐1,3,4‐oxadiazol‐2‐ylamino) ethyl 2‐naphthol[2,1‐*b*] furan‐1‐ylacetate (Figure 1) was discovered as an LTA synthesis inhibitor following an extensive small molecule library screen. Enzyme–substrate binding analysis revealed that compound 1771 abolished the interaction of the extracellular catalytic domain of LtaS (eLtaS) to PG and the subsequent cleavage of PG by eLtaS (Richter et al., 2013). Structure activity relationship (SAR) analysis and hydrophilic–hydrophobic molecular mapping showed compound 1771 bears structural similarity to PG and may act as a competitive inhibitor, disrupting LtaS‐mediated GroP polymerization. Compound 1771 has subsequently undergone a multitude of structural reconfigurations, directed by SAR and molecular dynamic simulations, resulting in a compound (designated compound 4; Figure 1) with greater binding affinity to eLtaS, improved antibacterial activity and significant β‐lactam synergy (Wezen et al., 2022). However, there is debate within the scientific community as to whether eLtaS is the primary target of compound 1771. The work of Takashi et al previously hypothesized that the azo dye congo red might disrupt LTA biosynthesis after discovering its antimicrobial activity against strains lacking wall teichoic acids (Suzuki et al., 2012). Subsequently, Vickery et al developed an assay employing artificial proteoliposomes reconstituted with full length LtaS whereby LTA anchors to the membrane via PG and promotes the synthesis of the polymer (Vickery et al., 2018). In this LtaS reconstitution assay only the azo dye Congo red resulted in enzymatic inhibition; compound 1771 had no LtaS inhibitory activity and therefore may inhibit LTA formation either by inhibiting one of the upstream essential LTA biosynthesis enzymes or through a more general toxicity mechanism.

A hybrid class of compounds inspired by Congo red and 1771, targeting LTA biosynthesis are the N‐(1,3,4‐oxadiazol‐2‐yl) benzamides, where one specific, highly active compound, HSGN‐94 (Figure 1) has been extensively characterized. HSGN‐94 has shown activity against multiple *S. aureus* and Gram‐positive strains, displaying low cytotoxicity, minimal in vitro resistance development following extensive passage and significant protection in *S. aureus* murine skin infection models (Naclerio et al., 2022). Interestingly, HSGN‐94 was found to directly interact with the cytoplasmic α‐phosphoglucomutase PgcA, an enzyme that functions at the beginning of the LTA pathway synthesizing glucose‐1‐phosphate from glucose‐6‐phosphate. In addition, HSGN‐94 exposure resulted in the downregulation of the multifunctional phosphatidylglycerol phosphatase synthase PgsA which is essential for PG synthesis and thus LTA polymerization (Naclerio et al., 2022).

Future work unravelling the precise drug target(s) for LTA inhibition will propel the clinical utility of these small molecule inhibitors where importantly the emergence of resistance following serial in vitro exposure has been very limited (Naclerio et al., 2022; Richter et al., 2013).

### FtsZ inhibitors

The filamentous temperature‐sensitive protein Z (FtsZ) is a major component of the divisome, a macromolecular complex that governs bacterial cytokinesis. FtsZ is the first protein to migrate to the site of division and self‐polymerizes in a GTP‐dependent fashion, forming protofilaments that aggregate into a filamentous ring‐like structure (Z‐ring). The Z‐ring serves as a cytoskeletal scaffold for the recruitment and organization of other cell segregation proteins that support septum formation and cell division (Han et al., 2021). FtsZ has recently emerged as an attractive antibacterial target for several reasons: (1) FtsZ is crucial for bacterial cell separation and survival; (2) FtsZ is highly conserved across bacterial species indicating that this protein would represent a broad‐spectrum antibacterial target; (3) FtsZ is not present in mammalian cells suggesting that FtsZ inhibitors would not pose significant toxicity concerns (Han et al., 2021; Tripathy & Sahu, 2019).

PC190723 represented a new class of benzamides that specifically targeted FtsZ (Figure 1). This molecule was discovered following an in‐depth fragment‐based approach using the small FtsZ ligand, 3‐methoxybenzamide as a scaffold. Although PC190723 exhibited impressive in vitro bactericidal activity against MRSA and demonstrated efficacy in a murine septicaemia model (Haydon et al., 2008), clinical development was hindered due to poor pharmacokinetic properties (Kaul et al., 2013). To circumvent these issues, two prodrugs of PC190723 (TXY436 and TXY541) were designed resulting in enhanced physiochemical qualities suitable for clinical administration but these molecules continued to suffer from poor pharmacokinetics (Kaul et al., 2013). A third prodrug (TXA709) of an alternative benzamide (TXA707) was developed which exhibited significantly increased metabolic stability, enhanced pharmacokinetic features and greater in vivo potency against *S. aureus* (Kate et al., 2015). The substitution of a Cl group with an electron‐with‐drawing CF_3_ functionality on the pyridyl ring of the prodrug conferred increased resistance to cytochrome P450‐mediated metabolism, preventing rapid elimination and extending the half‐life of the active product TXA707 (Figure 1, Kate et al., 2015). TXA709 is currently undergoing phase I clinical trials, as an oral formulation targeting MRSA infections (TAXIS, 2020).

The selection of TXA709 resistant mutants in vitro occurred at a frequency of 3 × 10^−8^ consistent with compounds that target a single gene product (Haydon et al., 2008). The resistance mechanism was attributed to target site modifications within the interdomain cleft of FtsZ with mutations G196S or G193D most frequently observed (Haydon et al., 2008; Kate et al., 2015). In response, researchers developed a modified TXA707 which possessed greater structural flexibility (TXA6101; Figure 1) and retained activity against isolates harbouring FtsZ mutations that confer resistance to TXA707 (Fujita et al., 2017), offering key insights into the structural basis for the design of future FtsZ inhibitors. TXA6101 also displayed lower frequency of resistance in MRSA compared to TXA707. Crystallographic analysis revealed TXA6101 can induce conformational changes in the FtsZ binding pocket and crucially avoid steric clashes with Ser196 and Asp193 facilitating tighter binding to mutant FtsZ (Fujita et al., 2017).

In summary, benzamide‐based FtsZ inhibitors are novel bactericidal drugs which have shown in vivo efficacy in animal models and can be modified to overcome resistance developed following intensive selection. Future clinical trials will report on the safety, tolerability, immunogenicity and efficacy, of FtsZ inhibitors either as single agents or in combination with clinically relevant antibiotics.

### FabI inhibitors

The bacterial fatty acid biosynthesis (FAS) pathway provides essential precursors for the formation of several important cellular components such as phospholipids and represents a primary step in membrane lipid biogenesis. In humans, the type I FAS pathway is governed by a single multifunctional enzyme complex with several distinct domains. Importantly in bacteria, the type II FAS pathway is controlled by distinct enzymes, one of which, the NADPH‐dependent enoyl‐acyl carrier protein (ACP) reductase FabI, has been shown to be essential for *S. aureus* viability (Campbell & Cronan, 2001; Ji et al., 2004). FabI functions during the last step of the biosynthetic pathway, controlling acyl chain elongation for saturated and unsaturated fatty acid synthesis (Campbell & Cronan, 2001). In *S. aureus* FabI is the only enoyl‐ACP reductase and is therefore essential. Combined with the organizational and structural difference in FAS between prokaryotes and eukaryotes, inhibitors of FabI represent a novel and promising anti‐staphylococcal target.

Two FabI inhibitors that have potent activity against multidrug resistant S*. aureus* have been extensively examined. Afabicin desphosphono (Debio 1452; AFN‐1252) and its corresponding prodrug Afabicin (Debio 1450; AFN‐1720) represent a first‐in‐class FabI inhibitor, composed of a 3‐methylbenzofuran and an oxotetrahydronaphthyridine linked by an N‐methylpropenamide (Figure 1). Afabicin demonstrates narrow spectrum inhibitory activity against staphylococci, with an MIC_90_ ≤ 0.015 μg/mL against clinically relevant MSSA and MRSA isolates (Karlowsky et al., 2009) and demonstrated superior activity over linezolid in a mouse model of septicaemia (Kaplan et al., 2012). Extensive biochemical analysis, macromolecular incorporation studies and crystallographic data confirmed FabI as the selective target for afabicin (Kaplan et al., 2012). A phase II multicentre trial demonstrated that afabicin desphosphono is well tolerated and was effective in the treatment of staphylococcal acute bacterial skin and skin structure infections (ABSSSIs) (NCT01519492) (Hafkin et al., 2016). Furthermore, Afabicin was shown to be non‐inferior to vancomycin/linezolid in the treatment of ABSSI in a phase II, randomized double blind multicentre study (NCT02426918) (Wittke et al., 2020).

Nilofabicin (CG400549) is a benzyl‐pyridone (Figure 1) which also exhibits potent activity against MSSA and MRSA isolates and was effective in combating systemic *S. aureus* infection in a murine model (Park et al., 2007). Overexpression of *fabI* in *S. aureus* resulted in enhanced resistance to nilofabicin. In addition, genome analysis of in vitro selected nilofabicin‐resistant *S. aureus* identified mutations in FabI (F204L) (Park et al., 2007) confirming the enoyl‐ACP reductase as the primary antibacterial target. A phase IIa open‐label exploratory study demonstrated safety and efficacy in patients with complicated ABSSSI (NCT01593761).

## ENGINEERED ANTIMICROBIAL PEPTIDES

Antimicrobial peptides (AMPs) are central components of the innate immune system and act as a first line of defence against microbial intruders. AMPs that target and permeabilize the bacterial membrane have long been viewed as potential alternatives to antibiotics. Their mechanism of action is hypothesized to engage nonspecifically with the membrane surface of pathogens and not individual proteins, which may be beneficial in reducing the emergence of resistance during treatment (Wimley & Hristova, 2011). AMPs act rapidly to compromise microbial membrane integrity and have been shown to act synergistically with clinically relevant antibiotics (Steenbergen et al., 2009; Wimley & Hristova, 2011). In this section we will review the most promising novel AMPs that have shown anti‐staphylococcal activity in recent clinical trials.

### PLG0206

PLG0206 (WLBU2) is an engineered 3.4 kDa, 24 residue cationic amphiphilic AMP (Huang, Brothers, et al., 2022). This AMP has been designed to have improved toxicity and maintain activity in the presence of physiologic concentrations of ions and acidic pH, both common limitations of naturally occurring AMPs (Wimley & Hristova, 2011).

PLG0206 has broad spectrum of activity, inhibiting numerous antimicrobial resistant priority pathogens, including *S. aureus*, when examined using in vitro and ex vivo inhibitory assays and in vivo animal models of infection (Huang, Brothers, et al., 2022). This AMP has shown significant inhibitory activity against mature *S. aureus* biofilms grown on metal implant material and in animal models designed to represent periprosthetic joint infections (PJIs) (Mandell et al., 2020). Detailed mechanistic dissection has shown that PLG0206 interaction with bacterial membranes result in lipid phase consolidation leading to local membrane stiffening and a loss of lipid order and membrane thickness. This results in lipid headgroup mismatches and leads to decreased ion flow (Heinrich et al., 2020). Furthermore, PLG0206 has undergone comprehensive non‐clinical safety studies which directed a phase I intravenous clinical safety study in healthy human volunteers, which highlighted an acceptable safety profile (NCT05137314) (Huang, Dobbins, et al., 2022). The developer, Peptilogics, plans to start phase II trials of PLG0206 as a treatment for PJIs of which *S. aureus* is major contributor.

### Brilacidin

Brilacidin (PMX30063) is a small molecule arylamide AMP mimic, composed of a planar scaffold decorated with two trifluoromethane hydrophobic substitutions and four positive guanadinyl and pyridinyl substitutions that was optimized for its activity against *S. aureus* (Choi et al., 2009). Treatment of *S. aureus* with brilacidin causes rapid depolarization of the membrane comparable to the clinically used lipopeptide antibiotic daptomycin. Further transcriptional profiling showed that like daptomycin, brilacidin induced the expression of cell wall stress responsive two‐component systems WalRK and VraRS, strongly indicating adverse effects on cell wall dynamics (Mensa et al., 2014).

Brilacidin underwent two randomized phase II clinical trials (NCT02052388 and NCT01211470) for the treatment of ABSSSI caused by *S. aureus,* using daptomycin as the comparator agent (Jorgensen et al., 2015). The primary endpoint of early clinical response defined as a >20% reduction in lesion size was high (>90%) in patients treated with brilacidin and comparable to the daptomycin‐treated group. No serious adverse events were documented indicating an appropriate safety profile. The developer, Innovation Pharmaceuticals, has since announced that they seek to start phase III clinical trials in patients with ABSSSI.

### OP‐145

OP‐145 (previously known as P60.4Ac) is a synthetic derivative of the human cathelicidin LL‐37, constructed to maintain antimicrobial activity towards Gram‐negative and Gram‐positive bacteria while limiting the proinflammatory activity of LL‐37. OP‐145 also contains modifications favouring amphipathic helix formation and alterations at the N‐ and C‐terminus to protect against proteolytic degradation and enhanced in vivo stability (Nell et al., 2006). OP‐145 has shown to be effective against MRSA using in vitro inhibitory assays, biofilm models and MRSA infected thermally wounded human skin equivalents, while maintaining low toxicity on human epidermal models (Haisma et al., 2014). OP‐145 has shown promise as a local topical therapeutic, retaining activity in formulations suitable for skin and nasal mucosa application (Haisma et al., 2016).

Investigation is currently underway for the suitability of OP‐145 as an ototopical solution for the treatment of chronic suppurative otitis media (CSOM). Recent data from a randomized, double‐blinded placebo‐controlled multicentre phase II study indicate that application of OP‐145 to ear canal of patients suffering from CSOM was safe and well‐tolerated. Otoscopic scoring used as a clinical endpoint demonstrated a higher treatment success in patients treated with OPA‐145 than in the placebo group (Peek et al., 2020).

### LTX‐109

LTX‐109 is another compound inspired by a naturally occurring peptide, drawing inspiration from lactoferricin. LTX‐109 comprises both cationic units and lipophilic groups incorporating nongenetically coded synthetic amino acids, a modified tryptophan residue and capped by an ethylphenyl group at the C‐terminus (Isaksson et al., 2011). This synthetic peptidomimetic displays fast‐acting, broad‐spectrum bactericidal antimicrobial activity, including inhibitory activity against MRSA and vancomycin‐resistant *S. aureus* (VRSA), and is less susceptible to host endoproteases (Isaksson et al., 2011; Saravolatz et al., 2012). To date no in vitro cross resistance with other AMPs or antibiotics has been observed, potentially because of the ultra‐rapid membrane destruction activity of LTX‐109 resulting in a lower likelihood for resistant mutants to emerge.

LTX‐109 has shown efficacy in phase I/IIa clinical trials assessing nasal decolonization in persistent MRSA and MSSA carriers (NCT01158235). Patients were treated for 3 days with placebo or 1%, 2% or 5% LTX‐109; a statistically significant decrease in *S. aureus* burden measured by CFU in nasal swabs was observed in subjects treated with 2% or 5% LTX‐109 (Nilsson et al., 2015). Systemic exposure was very low, and no safety concerns were reported following a 9‐week period. A phase II, double‐blind, placebo‐controlled, randomized study to examine the tolerability and efficacy of LTX‐109 administered topically to carriers of *S. aureus* was completed in 2021 (NCT04767321), however, the results have not been published.

## NEW NATURALLY OCCURRING ANTIBIOTICS

Historically, naturally occurring products derived from cultivable soil microorganisms have been the most important source of clinically used antibiotics. Recently, there has been renewed focus on the discovery of naturally produced compounds following advancements in techniques designed to isolate and propagate previously unculturable microorganisms. Innovative technologies such as membrane diffusion‐based reactors, microfluidic‐based cultivation systems and cell‐sorting technologies coupled with genome‐resolved metagenomic approaches have the potential to drive a new era in antibiotic drug design and development (Lewis et al., 2021). Below, we have discussed promising examples of naturally occurring antibiotics with anti‐staphylococcal activity.

### Teixobactin

**FIGURE 2:**
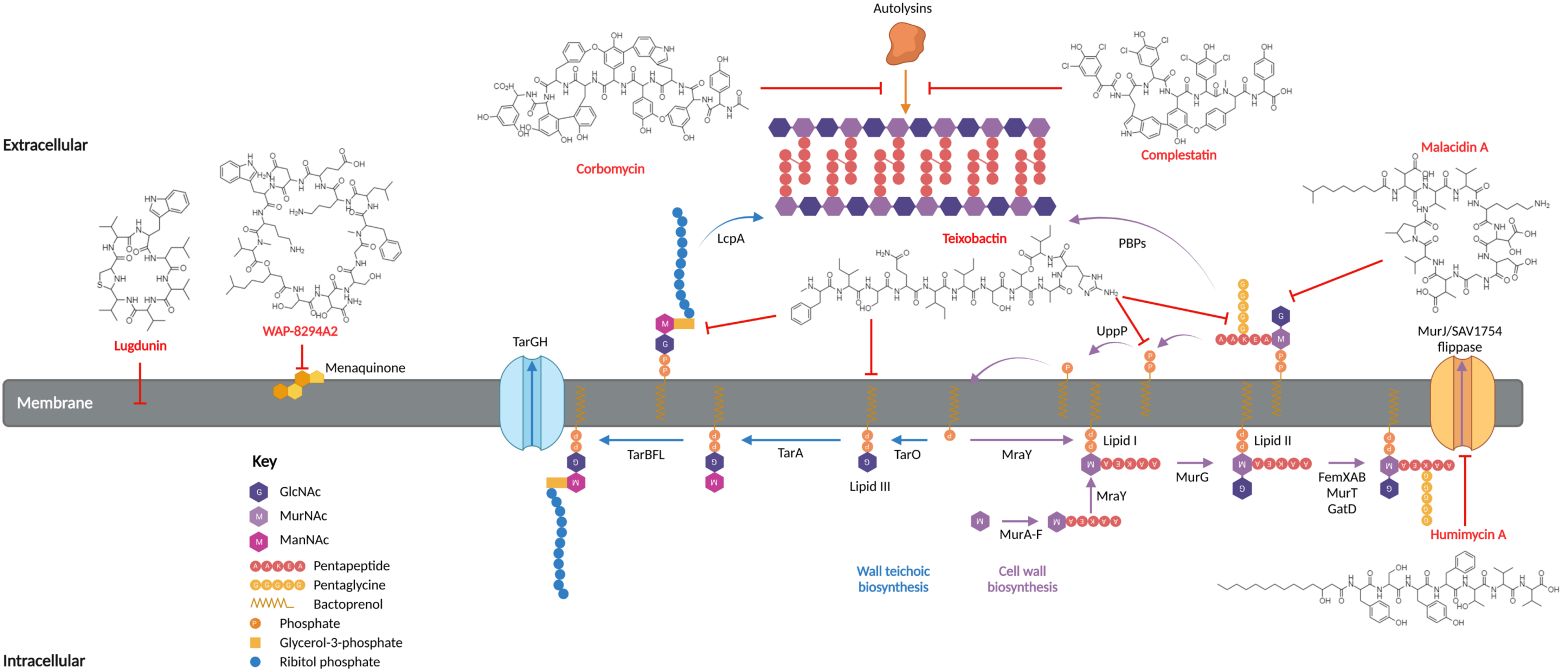
Structure and mechanism of action of new naturally occurring antibiotics. Cell wall synthesis begins with the formation of UDP‐MurNAc action through the action of MurA and MurB. The pentapeptide is subsequently added to UDP‐MurNAc acid by the enzymes MurC‐F to form the phosphor‐MurNAc‐pentapeptide. This is ligated to the lipid carrier undecaprenyl‐diphosphate by MraY to form the lipid I precursor. The addition of UDP‐GlcNAc by MurG finally results in the formation of lipid II. FemX then adds the first glycyl unit, FemA the second and third, and FemB the fourth and fifth to complete the addition of the pentaglycine cross bridge. Final structural modification of lipid II, such as deamination of D‐Glu on the stem peptide, is performed by MurT and GatD. The completed lipid II molecule is subsequently flipped to the outer membrane through the action of MurJ/SAV1754 which is blocked by the action of humimycin A and humimycin B. The final stage of cell wall synthesis is carried out by penicillin‐binding proteins (PBPs), which incorporate lipid II into the growing peptidoglycan sacculus through transglycosylation and transpeptidation reactions. This is prevented through the action of malacidin A and teixobactin which bind to extracellular lipid II and prevent PBP binding. As peptidoglycan matures it undergoes structural reconfiguration through the action of autolysins. The binding of complestatin and corbomycin to conserved peptidoglycan residues prevents the binding of autolysins thus inhibiting cell wall turnover. After the addition of lipid II into the cell wall, undecaprenyl diphosphate is recycled to undecaprenyl phosphate by UppP and used as a precursor for wall teichoic synthesis. TarO and TarA catalyse the formation of the disaccharide linker which is conjugated to undecaprenyl diphosphate. Next two glycerol phosphate residues are added by TarB which sets the stage for teichoic acid polymerization. Repeating subunits of ribitol phosphate are added by TarF and TarL. The completed teichoic acid is flipped to the outer membrane by TarGH and incorporated in the cell wall by the teichoic ligase LcpA. Importantly, teixobactin has been shown to bind to multiple cell wall and teichoic acid precursors including lipid III and undecaprenyl diphosphate and lipid I. WAP‐8294A2 is thought to cause menaquinone‐dependant membrane lysis. Lugdunin is thought to cause membrane disruption through an unknown mechanism of action.

Teixobactin is a hugely promising new cell wall targeting antibiotic currently in preclinical stages of development. Unearthed using the diffusion‐based isolation (i)CHIP technology, teixobactin was isolated from the β‐proteobacteria, *Eleftheria terrae* and displayed broad‐spectrum activity against the multidrug resistant Gram‐positive organisms, *S. aureus, Streptococcus pneumoniae* and vancomycin resistant enterococci (VRE) and in vivo efficacy in multiple animal models of infection (Ling et al., 2015). Structurally, this compound is an undecapeptide consisting of five non‐canonical amino acids which includes four D‐amino acids and a cationic L‐*allo*‐enduracididine located in a C‐terminal depsi‐cycle (Figure 2). Using a combination of solid‐state NMR, confocal microscopy and molecular dynamic simulations, the mechanism of activity of this molecule was resolved at the atomic level. The C‐terminal enduracididine motif enables teixobactin to bind to the pyrophosphate‐sugar moiety of lipid II, while the N‐terminus facilitates oligomerization of multiple teixobactin molecules with other lipid II molecules promoting the configuration of a β‐sheeted teixobactin/lipid II macromolecule that forms an intricate fibrillar structure (Figure 2, Shukla et al., 2022). These fibrils not only obstruct peptidoglycan synthesis, but the hydrophobic tails of lipid II aggregate within the supramolecular fibrillar structure, displacing phospholipids resulting in membrane defects. Lastly, teixobactin has also been shown to target a conserved motif on lipid III a precursor of cell wall teichoic acids; thus, this molecule causes extensive damage to the cell envelope, halting peptidoglycan synthesis and disrupting membrane integrity (Ling et al., 2015; Shukla et al., 2022). Importantly, the highly conserved pyrophosphate‐sugar moiety of lipid II cannot be readily altered, thus precluding the development of target site resistance. However, it is possible that enzymatic drug modification systems have developed in competitor soil bacterium.

### Complestatin and corbomycin

A phylogeny‐guided analysis of glycopeptide antibiotic biosynthetic gene clusters (BGCs) that lacked known self‐resistance genes identified two compounds with potent anti‐staphylococcal activity (Culp et al., 2020). One of the analysed BGCs contained the known compound complestatin produced by *Streptomyces* sp. WAC01325. The second compound was derived from BGCs sourced from a divergent clade with no known characterized members. Phylogenetic based structural predictions enabled the isolation of a metabolite from *Streptomyces* sp. WAC01529 which was subsequently named corbomycin (Culp et al., 2020).

Both complestatin and corbomycin (Figure 2) were found to be active against MRSA, daptomycin resistant *S. aureus*, vancomycin intermediate *S. aureus* (VISA), and VRE, indicating a different mechanism of action compared to classical glycopeptide antibiotics. Exposing the model Gram‐positive organism, *Bacillus subtilis,* to sub‐MICs of both compounds showed significant peptidoglycan dysfunction, distorted septal formation, thickened cell wall and granular formations in the cytoplasm, similar to *B. subtilis* mutants defective in autolysin production. Using imaging, biochemical and whole‐cell systems, complestatin and corbomycin were observed to inhibit the activity of diverse peptidoglycan hydrolases and treatment with sub‐MICs resulted in the accumulation of peptidoglycan (Culp et al., 2020). The results suggest that both compounds interfered with the activity of autolysins, enzymes central in peptidoglycan remodelling and cell division (Vollmer et al., 2008).

As both molecules could inhibit a broad range of structurally unrelated autolysins, it was suggested that they bind to the autolysin substrate, peptidoglycan. Indeed, pretreatment of purified peptidoglycan with both molecules prevented digestion by the *B. subtilis* cell wall endopeptidase. Further work using BODIPY labelled complestatin and corbomycin showed that these compounds bound conserved residues within the peptidoglycan structure, supporting their broad‐spectrum activity. Combined, these results indicate a novel mechanism of action whereby complestatin and corbomycin bind to peptidoglycan and interfere with the ability of autolysins to recognize their substrate, blocking peptidoglycan modelling (Figure 2). Excitingly, both molecules were shown to be effective in treating *S. aureus* superficial skin infection in a neutropenic mouse model with similar efficacy to the topical antibiotic fusidic acid (Culp et al., 2020).

### Malacidins

The strategy that resulted in the discovery of complestatin and corbomycin has also been applied to search the metagenomes of 2000 soil samples for the presence of lipopeptide BCGs with calcium‐binding motifs. Subsequent phylogenetic analysis showed numerous divergent clades not associated with known lipopeptide BCGs. One distinct clade was found in 19% of metagenomes, suggesting the presence of an abundant, previously uncharacterized family of calcium‐dependant antibiotics which the authors christened as malacidins (Hover et al., 2018). Recovery of the malacidin specific BCG was achieved through the construction of an *E. coli* cosmid library using purified DNA from a desert soil that was rich in the malacidin natural product sequence tag. Compound analysis revealed that malacidins were cyclic lipopeptides with a protein core comprised of four non‐canonical/proteinogenic amino acids (Figure 2). Malacidins lack the known Asp‐X‐Asp‐Gly calcium‐binding motif, however, in vitro susceptibility testing across a range of calcium ion concentrations showed a clear dependency on calcium for antibacterial activity, confirming the presence of an alternative calcium‐binding motif. Malacidins have a spectrum of activity which includes a broad range of Gram‐positive pathogens including multidrug resistant (MDR) *S. aureus* strains and VRE with an MIC of 0.1–2 μg/mL. In vivo potency of malacidins has been demonstrated in a rat wound model in which malacidin A was topically administered. Following 24 h and 72 h postinfection, malacidin A was successful in significantly reducing wound bacterial burdens. Likewise, the authors noted no substantial toxicity or haemolytic activity even at concentrations >100× the MIC (Hover et al., 2018). A known drawback of daptomycin as a therapeutic agent is its inability to treat bacterial pneumonia due to being inactivated by pulmonary surfactant (Silverman et al., 2005). However, malacidins do not share this encumbrance allowing them to be further investigated for this type of infection (Hover et al., 2018).

Treatment with malacidin A results in accumulation of the cell wall precursor molecule undecaprenyl‐*N*‐acetylmuramic acid‐pentapeptide (Figure 2), which is consistent with other cell wall targeting antibiotics such as vancomycin and friulimicin (Reynolds, 1989). Further thin layer chromatography analysis using a mobility shift assay found malacidin to be a lipid II binder. The lipid II binding was found to be distinct as malacidins retain activity against vancomycin resistant pathogens. The authors have also noted that *S. aureus* seems unable to induce resistance against malacidin, with no notable shift in MIC over 20 days of passage (Hover et al., 2018). Whether resistance can be achieved through horizontal gene transfer from soil competitors requires further study.

### Lugdunin

Lugdunin is the first reported non‐ribosomally synthesized bioactive compound from human microbiomes. Produced by *Staphylococcus lugdunensis*, a nasal and skin commensal, lugdunin is the founding member of a novel class of thiazolidine‐containing cyclic peptide antibiotics (Figure 2, Zipperer et al., 2016). This molecule possesses potent bactericidal activity against a broad range of Gram‐positive pathogens, including MRSA. Moreover, lugdunin was shown to be effective in reducing *S. aureus* burden in a mouse skin infection model. Furthermore, the presence of *S*. lugdunensis in the human nares interferes with *S*. *aureus* carriage, reducing colonization by approximately sixfold compared to persons not colonized by the commensal (Zipperer et al., 2016).

It is thought that lugdunin exerts its bactericidal effect on *S*. *aureus* through membrane potential dissipation in a protonophore‐like mechanism (Schilling et al., 2019). In addition to its bactericidal action, lugdunin exerts immunomodulatory activity by stimulating human skin cells to produce synergistically acting host defence peptides (Bitschar et al., 2019), offering a multi‐level protection against *S. aureus*. Promisingly, in vitro serial passage experiments noted a low risk for the emergence of resistance by mutation, however, it was observed that *S*. *lugdunensis* was not susceptible to the antimicrobial function of lugdunin, suggesting a self‐resistance mechanism. The *lugEFGH* genes located adjacent to the lugdunin BGC were recently discovered to encode ABC transporters which mediate lugdunin secretion and confer resistance to the antibiotic (Krauss et al., 2020). LugEF was shown to have a dominant role in lugdunin release whereas LugGH acts as the central self‐resistance transporter. The substrate specificity of both ABC transporters was observed to be largely specific to lugdunin (Krauss et al., 2020). This has clinical relevance if lugdunin were to be administered as a therapy; structural variants of lugdunin, some of which have been observed to have higher anti‐staphylococcal activity than native lugdunin, would not be neutralized by Lug transporters if the *lugEFGH* operon did spread horizontally to other bacterial species such as *S*. *aureus* (Krauss et al., 2020).

### Humimycins

The human microbiome has further proved to be an invaluable, untapped resource of antimicrobials through the discovery of humimycins. Genomes of human commensal and pathogenic bacteria were screened for the presence of gene clusters that encoded large non‐ribosomal peptides (NRPs). This led to the discovery of 57 unique putative non‐ribosomal peptide synthases (NRPS). Subsequent screening of the peptide products against various commensal and pathogenic species for antibacterial activity found two antibiotics, playfully named humimycin A and humimycin B after h uman microbiome mycin, from *Rhodococcus* spp. (Figure 2). Both humimycins were found to have broad activity against Firmicutes and some limited activity against Actinobacteria. The most impressive potency was found when tested against *S. aureus* and *S. pneumoniae* with MICs of 8 μg/mL and 4 μg/mL respectively. When tested against a panel of *S. aureus* clinical isolates, MICs of 8–128 μg/mL were reported. In an effort to elucidate the biochemical mode of action, *S. aureus* USA300 mutants were selected that could grow in the presence of 2.5× the MIC. Pairwise bioinformatic analysis of 23 resistant strains found that they all contained a non‐synonymous mutation in the essential gene SAV1754 (SAUPAN004432000 pangenome locus tag). Of these 23 resistant mutants, 15 contained no other mutations. To investigate the involvement of SAV1754 in the resistance to humimycins, overexpression studies were performed. Overexpression of SAV1754 conferred elevated MICs against humimycin A of >128 μg/mL, confirming its involvement (Chu et al., 2016). SAV1754 is believed to encode a homologue of the MurJ lipid II flippase. While MurJ is essential in many species of bacteria, limited studies have investigated its potential as an antibacterial agent. However, a small molecule screen for molecules that could reverse β‐lactam resistance, identified synthetic inhibitors of SAV1754 (Huber et al., 2009; Lee et al., 2016). Humimycin A was subsequently evaluated for β‐lactam potentiation and was found to reduce the dicloxacillin MIC from 256 μg/mL (resistant) to 8 μg/mL (sensitive) in the presence of 4 μg/mL humimycin A. Further investigation of this combination in a mouse peritonitis sepsis model found that treatment with dicloxacillin/humimycin A significantly improved the survival rate compared with treatment of dicloxacillin or humimycin A alone (Chu et al., 2016).

### Cyclic lipopeptide

The natural metabolite, WAP‐8294A2 (also called lotilibcin), was first isolated from the culture broth of the environmental Gram‐negative bacterium *Lysobacter staphylocidin* and belongs to a promising class of cyclic lipodepsipeptide antibiotics (Figure 2) (Kato et al., 1997). Structurally, it contains a 40‐membered macrocycle comprising 12 amino acid residues linked by a small hydroxylated fatty acid (Kato et al., 1997). This molecule possesses potent and rapid bactericidal activity against *S. aureus* and VRE, however, *S. aureus* mutants deficient in menaquinone, an essential coenzyme for electron transfer in bacterial respiration, were resistant. Recent mechanistic studies using unilamellar vesicles containing menaquinone or not, confirmed that WAP‐8294A2 causes membrane lysis in a menaquinone‐dependent fashion (Itoh et al., 2018). WAP‐8294A2 displays impressive potency towards MRSA (1 μg/mL MIC) and has shown to be efficacious in a systemic mouse infection model (Itoh et al., 2018). In 2011 WAP‐8294A2 entered phase I/II trials for the treatment of acne vulgaris in the United States. As of 2017, there have been no reports on the subsequent development of topical WAP‐8294A2 although it is also in preclinical development as an IV infusion in Japan (Butler & Paterson, 2020).

## ANTIBIOTIC ADJUVANTS

Antibiotic adjuvants represent an alternative strategy to developing new drugs to replace antibiotics rendered obsolete due to resistance. In this setting, resistance is bypassed by coupling antibiotics with specific adjuvants designed to target the resistance element. Prolonging the life of antibiotics by using antibiotic‐adjuvant combinations has proved to be incredibly successful. For example, the use of broad‐spectrum β‐lactamase inhibitors (such as clavulanic acid) that target bacterially produced β‐lactamases that function to hydrolyse the β‐lactam ring, has worked to significantly curtail resistance and increase treatment options (Bush & Bradford, 2016). Specific antibiotic adjuvants that are being developed against *S. aureus* are described below and summarized in Table 1.

**TABLE 1:** Antibiotic adjuvants and β‐lactam potentiators.

Compound	Description	Antibiotic partner
Antibiotic adjuvants		
Nilotinib	NorA efflux pump inhibitor	Ciprofloxacin
PS‐ODN04	Antisense *mecA* oligonucleotide	Oxacillin
M‐C7.1	MprF targeting antibody	Daptomycin
β‐lactam potentiators		
Statins	Defective PBP2a oligomerization	β‐lactams
Polyamines	Possible inhibition of PBP2	β‐lactams
Krysinomycin/actinocarbasin	Disruption of PBP2a secretion through inhibition of signal peptidase SpsB	β‐lactams
Purine nucleoside analogues	c‐di‐AMP disruption	β‐lactams

### Efflux pump inhibitors

Efflux pumps are active bacterial transport proteins embedded in the cytoplasmic membrane that function as defence systems, mediating the extrusion of toxic substrates from within bacteria to the environment. In. *S. aureus* there are over 30 chromosomally and plasmid‐encoded efflux pump genes that can confer resistance to numerous classes of antibiotics and biocides (Costa et al., 2013). Thus, inhibiting the activity of efflux pumps represents an exciting target for antibiotic adjuvants.

Perhaps the most well‐studied *S. aureus* efflux pumps are the Nor family which together can expel both hydrophobic and hydrophilic fluoroquinolones, chloramphenicol and several other structurally diverse compounds (Costa et al., 2013). The plant alkaloid reserpine has been shown to be an inhibitor of bacterial efflux pumps as well as eukaryotic multidrug efflux transporter systems (Ahmed et al., 1993). In *S. aureus,* reserpine acts as an efflux pump inhibitor (EPI), reversing fluoroquinolone resistance through the inhibition of NorA, however, concentrations required for inhibition are toxic to mammalian cells (Dashtbani‐Roozbehani & Brown, 2021). Since the discovery of reserpine, various other plant‐derived EPIs have been uncovered most of which belonging to the alkaloid, flavonoid, terpenoid and polyphenol class (Dashtbani‐Roozbehani & Brown, 2021). Despite their clear efficacy in restoring antimicrobial activity, none of these EPIs have progressed beyond the early stages of development due to their acute toxicity profiles. In response, current research in this field has centred on screening clinically approved drugs for efflux pump inhibition. Following molecular docking and virtual screening analysis against a NorA homologue, the tyrosine kinase inhibitor nilotinib was identified to possess potent efflux pump inhibitory activity (Zimmermann et al., 2019). Treatment with nilotinib resulted in a 16‐fold reduction of ciprofloxacin MIC and led to a significant reduction in preformed mature biofilms (Zimmermann et al., 2019). Nilotinib is used in the treatment of chronic myelogenous leukaemia; inhibition of NorA efflux pump activity occurred at significantly lower concentrations to that which is clinically achievable and tolerable, suggesting that this EPI is the first to achieve inhibition at sub‐toxic concentrations.

### Antisense *mecA*

The resistance of MRSA to practically all members of the β‐lactam family of antibiotics is mediated by *mecA*, the gene which encodes an alternative PBP2a. Unlike intrinsic *S. aureus* PBPs, PBP2a resists covalent modification by β‐lactam antibiotics at the enzyme active site and has a lower binding affinity to β‐lactams than native PBPs (Fuda et al., 2004). The *mecA* gene is highly conserved across *S. aureus* isolates making it an ideal target for therapeutic inhibition.

Antisense oligonucleotides (ASOs) offer a novel intervention strategy to combat infectious diseases. ASOs are relatively short (13–25 nucleotides), single‐stranded molecules that hybridize to a complementary mRNA, preventing the translation of the target gene (Dias & Stein, 2002). Structural modifications can be utilized to increase stability against intracellular nucleases and enhance interaction with complementary targets. An antisense *mecA* phosphorothioate oligonucleotide has been designed (PS‐ODN04) which targets a sequence between nucleotides 854 and 871 within the mRNA coding region of *mecA* (Meng et al., 2015). Clinical isolates treated with PS‐ODN04 regained their susceptibility towards oxacillin. PS‐ODN04 partnered with oxacillin also significantly lowered the bacterial titres and improved survival rates of mice infected with clinical MRSA isolates, compared to mice treated with oxacillin alone. A potential drawback of PS‐ODN04 is its liposomal packaging. Packaging of ASOs within liposomes can be inefficient and can be associated with toxicity and off target accumulation. Advances in this field have led to antisense *mecA* being packaged in multi‐layer coated gold nanoparticles (Beha et al., 2021). This nanoparticle delivery system exhibited low cytotoxicity, was preferentially taken into bacteria over mammalian cells and significantly reduced *mecA* expression in MRSA isolates, offering an attractive delivery system for ASOs anti‐resistance application.

### Antibody blocking of MprF

Multiple peptide resistance factor (MprF) is a bifunctional membrane protein, acting as both the synthase and flippase of the positively charged phospholipid lysyl—phosphatidylglycerol within the bacterial membrane. Importantly, MprF is a major contributor to resistance to cationic AMPs and the clinically used antibiotic daptomycin (Ernst & Peschel, 2011). Topological predictions indicate that MprF consists of 14 transmembrane segments linked by loops between 2 and 56 amino acids in sequence, some of which are exposed on the outer surface of the bacterial membrane (Ernst et al., 2015).

Using phage display libraires and cyclized peptides corresponding to specific loop domains of MprF, Slavetinsky et al developed a monoclonal MprF targeting antibody (M‐C7.1) which bound to a specific epitope in the flippase domain (Slavetinsky et al., 2022). Incubation with M‐C7.1 sensitized *S. aureus* to cationic AMPs and daptomycin and promoted killing by polymorphonuclear lymphocytes (Slavetinsky et al., 2022). Therefore, targeted inhibition of MprF represents a promising antibiotic adjuvant, not only helping to re‐potentiate an antibiotic mainstay of *S. aureus* therapeutics, but also promoting the capacity of innate defences to clear *S. aureus* infections.

A frequently encountered criticism of antibody dependant anti‐virulence/anti‐resistance strategies, is the ability of the antibody to transverse the thick cell wall and reach its target. While recent insights into *S. aureus* cell wall structure found large irregular pores which could potentially allow antibodies to pass through (Pasquina‐Lemonche et al., 2020), it is possible that cell‐wall remodelling could limit the effectiveness of M‐C7.1. In addition, the utility of M‐C7.1 on non‐*mprF* daptomycin‐resistance conferring mutants (Bayer et al., 2013) requires attention as the requirement of MprF in these alternative daptomycin resistance mechanisms is poorly understood.

## β‐LACTAM POTENTIATION

β‐Lactams represent the most successful class of antibiotics, owing to their broad spectrum of use, low toxicity, exceptional pharmacokinetics and often rapid bactericidal activity (Foster, 2019). Their use has been restricted in treating *S. aureus* infections due to the development of resistance following the acquisition of SCC*mec*. Importantly, correct localization of PBP2a requires a chaperone, the membrane bound PrsA foldase protein (Jousselin et al., 2015). In addition, intricate membrane platforms are central for the correct oligomerization and stability of PBP2a (Garcia‐Fernandez et al., 2017). Recent studies have identified molecules that potentiate β‐lactam activity (Table 1) through disrupting cell division machinery or destabilizing cell envelope scaffolds required for PBP2a function.

### Statins

Statins, widely used in the prevention of cardiovascular disease, act as selective and competitive inhibitors of 3‐hydroxy 3‐methyglutaryl coenzyme A (HMG‐CoA) reductase which catalyses the conversion of HMG‐CoA to mevalonate, a precursor of cholesterol (Shitara & Sugiyama, 2006). In *S. aureus*, statins have been shown to inhibit the synthesis of the antioxidant carotenoid pigment staphyloxanthin, targeting *S. aureus* HMG‐CoA (dehydrosqualene synthase) and enhancing the susceptibility of *S. aureus* to reactive oxygen species (Liu et al., 2008). Recent seminal work has identified a prokaryotic version of membrane lipid rafts termed functional membrane microdomains (FMM) where diverse bacterial processes are compartmentalized (Lopez & Kolter, 2010). FMM harbour isoprenoid membrane lipids such as staphyloxanthin, colocalized with flotillin homologue proteins, forming a platform for the assembly of multimeric protein complexes involved in signalling and transport. Importantly in MRSA, PBP2a, PBP2 and PrsA form part of the FMM cargo and FMM are crucial for correct PBP2a oligomerization, with PBP2a interacting with and colocalizing with flotillin in membrane foci (Garcia‐Fernandez et al., 2017). Importantly, flotillin mutants were observed to be defective in PBP2a oligomerization and *S. aureus* cells treated with the statin zaragozic acid, known to impact staphyloxanthin synthesis (Liu et al., 2008) compromised PBP2a integrity. Significantly, deletion of flotillin or treatment of bacteria with statins interfered with β‐lactam resistance and resulted in MRSA cells being more susceptible to β‐lactams in a murine systemic infection model (Garcia‐Fernandez et al., 2017).

### Polyamines

Polyamines are polycationic molecules composed of at least two primary amines separated by a hydrophobic chain. These molecules have numerous cellular functions; the uniform distribution of positive charges decorating the hydrophobic backbone permit interaction with polyanionic macromolecules such as nucleic acids, phospholipids and some proteins. Consequently, these molecules play diverse roles in gene expression, signal transduction, membrane stabilization, translation and immune modulation (Sakamoto et al., 2020; Tabor & Tabor, 1984). Polyamines were initially believed to be synthesized and required by all life forms, however, polyamine‐auxotrophic and polyamine‐independent bacterial species have been identified (Joshi et al., 2011). *S. aureus* is an atypical example, displaying bactericidal hypersensitivity to polyamines at concentrations that are beneficial to the growth of other bacterial species (Joshi et al., 2011).

Recent work has exploited *S. aureus* susceptibility to polyamines generating novel linear polyamines one of which, AHA‐1394, showing enhanced activity against multiple antibiotic resistant *S. aureus* strains including MRSA, VRSA and the polyamine resistant USA300 strain LAC (MIC_50_ 2 μg/mL); a greater than 128‐fold increase in inhibition compared to naturally occurring polyamines (Douglas et al., 2022). Toxicity of the novel linear polyamines was comparable to clinically used anti‐staphylococcal antibiotics. Resistance against polyamines was observed in vitro via mutation of the *mprF* gene, however, resistant mutants acquired a marked decrease in relative fitness and displayed increased sensitivity to oxacillin (Douglas et al., 2022). Other studies have noted that polyamines, specifically spermine and spermidine have been shown to increase the susceptibility of *S. aureus* and other pathogens to β‐lactam antibiotics (Kwon & Lu, 2007). Importantly, AHA‐1394 showed significant antibiotic potentiation with oxacillin reducing MIC values by 16–64‐fold. Although the exact mechanism responsible for polyamine β‐lactam synergy is not fully clear, studies have reported a truncated PBP2 mutant devoid of transpeptidase activity but retaining transglycosylase function exhibiting resistance to β‐lactams in the presence of spermine (Yao & Lu, 2012). This data implicates PBP2 or PBP2‐dependent multi‐enzyme complexes as targets for polyamines that may disrupt normal PBP localization and function.

### Signal peptidase inhibitors

*Staphylococcus aureus* signal peptidase IB (SpsB) is a serine protease that functions to cleave the N‐terminal signal peptide of secreted proteins, a requirement for protein export through the Sec and Tat secretion systems (Paetzel et al., 2002). Following a high‐throughput, β‐lactam potentiator screen, two naturally occurring, structurally distinct compounds, the depsipeptide krisynomycin and the lipoglycopeptide actinocarbasin were shown to potentiate the activity of imipenem at concentrations 16‐fold lower than the MIC (Therien et al., 2012). Fitness test profiling, an assay where strains harbouring inducible antisense RNA oligonucleotides specific to essential genes are challenged with compounds, revealed SpsB to be the specific target of krisynomycin and actionocarbasin. Synergy was also shown for an extended panel of β‐lactams but not observed for other antibiotic classes, indicating that SpsB inhibitors may prevent the secretion of proteins required for β‐lactam resistance, such as PBP2a which possess a canonical N‐terminal leader sequence. A synthetic derivative of actinocarbasin termed M131, which contained improved activity and pharmacological properties also exhibited synergism with imipenem and was shown to be effective in vivo against MRSA in murine deep tissue and systemic infection models (Therien et al., 2012). Recent work has identified the importance of the halogenation pattern on the antimicrobial activity and imipenem enhancing effects of krisynomycin (Perez‐Bonilla et al., 2020), which may promote clinical application of signal peptide inhibitors in combination therapy with β‐lactam antibiotics.

### Synergy with purine nucleoside analogues

Nucleotide‐based signalling molecules facilitate the rapid coordination of cellular pathways that help bacteria adapt to changes in their environment (Grundling & Lee, 2016). Interestingly, when *S. aureus* is exposed to β‐lactam antibiotics, mutations independent of the *SCCmec* element that confer high‐level β‐lactam resistance have been observed. Upon examination these mutations have been primarily linked to the induction of the stringent stress response (Kim et al., 2013) and associated with enhanced levels of the nucleotide second messenger cycli‐di‐AMP (Corrigan et al., 2011). This association between increased purine signalling molecules, (p)ppGpp and c‐di‐AMP, and enhanced resistance to β‐lactams implicate purine metabolism in the regulation of β‐lactam resistance in MRSA and represent a target for β‐lactam potentiation. Recent work has shown that exposure of MRSA to purine nucleoside guanosine and xanthosine significantly reduce resistance to β‐lactams (Nolan et al., 2022). These nucleotides which are shunted through the GTP arm of purine biosynthesis, cause a significant decrease in c‐di‐AMP levels and a dramatic increase in cell size, particularly in the presence of oxacillin. Moreover, *S. aureus* strains deficient in the c‐di‐AMP phosphodiesterase GdpP, which functions to breakdown c‐di‐AMP could not be rendered sensitive to oxacillin upon guanosine exposure (Nolan et al., 2022). Accordingly, purine nucleotides that disrupt c‐di‐AMP regulation act as potent β‐lactam adjuvants, resensitizing MRSA to clinically susceptible concentrations of β‐lactam antibiotics. Importantly, compounds derived from nucleotides are clinically used to treat cancer and certain viral infections, illustrating their therapeutic potential to combat MRSA disease.

## ANTIBIOTIC‐ANTIBODY CONJUGATES

Although classically considered an extracellular pathogen, research over the last decades has confirmed that *S. aureus* frequently invades mammalian cells where it can survive, replicate and persist intracellularly (Fraunholz & Sinha, 2012). *S. aureus* invasion of host cells offers shelter against complement, neutralizing antibodies, circulating phagocytic immune cells a AMPs (Fraunholz & Sinha, 2012) and protection from clinically relevant anti‐staphylococcal antibiotics (Lehar et al., 2015). Failure to eradicate intracellular bacteria is associated with chronic and recurrent disease (Fraunholz & Sinha, 2012; Lehar et al., 2015).

Antibody–antibiotic conjugates (AACs) are potent therapeutics designed to specifically eliminate intracellular reservoirs of *S. aureus*. DSTA4637S is a specific AAC consisting of an engineered monoclonal human anti‐*S. aureus* IgG that specifically recognizes teichoic acid β‐*O*‐linked *N*‐acetylglucosamine sugars. This antibody has been designed to contain unpaired cysteines residues (THIOMAB™) permitting the covalent linkage to a rifamycin derivative (rifalogue; dmDNA31) via a cathepsin‐cleavable linker with the resulting compound possessing a stoichiometry ratio of two rifamycin molecules to one IgG. DSTA4637S has been shown to opsonize bloodstream‐circulating MRSA efficiently promoting uptake by phagocytic immune cells. Following phagocytosis, intracellular proteases cleave the linker and release high concentrations of the active bactericidal form of the antibiotic, eliminating intracellular *S. aureus* (Lehar et al., 2015). This AAC was shown to be more effective than vancomycin in a murine bacteraemia model, specifically when treatment with either AAC or vancomycin was initiated 24 h after infection.

A randomized, double‐blind, placebo‐controlled single‐ascending‐dose phase I trial was conducted to determine the safety and pharmacokinetics of DSTA4637S in healthy volunteers (NCT02596399). A favourable pharmacokinetic profile and no safety concerns were observed following single dose intravenous injection (Peck et al., 2019).

## BACTERIOPHAGE AND PHAGE LYSINS

### Bacteriophage therapy

A promising alternative to antibiotics is the use of bacterio(phages), viruses that infect and multiply inside bacterial cells. Virulent phages that undergo a lytic life cycle have promising therapeutic potential due to the deployment of phage‐encoded lysins that degrade the bacterial cell wall mediating cell destruction and subsequent viral release (Kakasis & Panitsa, 2019). A main advantage of phage therapy over conventional strategies is the precision and narrow spectrum of phage targeting specific pathogens, thus minimizing damage to the human microbiota (Kakasis & Panitsa, 2019). Due to increasing health issues caused by AMR, research into phage therapy has undergone a significant resurgence in recent years. This is in part due to the compassionate (sanctioning the use of unapproved drugs or therapies to combat disease when all available conventional therapies have failed) use of phages, which have largely been associated with positive clinical outcomes and a high degree of safety (Plumet et al., 2022). These positive results have spurred an increase in phage therapy‐related clinical investigations to combat *S. aureus*.

In recent years, two completed clinical trials have reported efficacy in the treatment of *S. aureus* infection using phage. The first, completed in 2009, investigated the use of the phage cocktail WPP‐201 against venous leg ulcers. At the end of the 12‐week dosing regime, safety of the phage cocktail was confirmed, and the therapy did not deleteriously affect wound healing (Rhoads et al., 2009). The next clinical trial came a decade later and investigated the use of the AB‐SA01 phage cocktail for the treatment of chronic rhinosinusitis caused by *S. aureus*. The phage therapy was well tolerated, with no fatalities recorded and the infection eradicated in two of nine patients (Ooi et al., 2019). Three further clinical trials are ongoing: a phase I study of phage therapy in combination with antibiotics for the treatment of PJIs of the knee or hip (NCT04787250), a phase I study of the SPK phage cocktail in the prevention of infections in second degree burn patients (NCT04323475), and finally a phase I study of a topical phage solution for the treatment of diabetic foot ulcers (NCT02664740).

Although phage therapy is considered a promising alternative to antibiotics, it is still in its infancy and several obstacles related to safety and immunogenicity, manufacturing and regulation (Dabrowska, 2019; Wittebole et al., 2014), need to be addressed before being used as an alternative to antimicrobial chemotherapy.

### Lysins

Phage‐encoded lysins are enzymes expressed during the final stage of lytic phage replication which specifically target and hydrolyse the bacterial cell wall peptidoglycan (Vazquez et al., 2018). Lysins have numerous advantages over classical antibiotics: (1) minimal off‐target damage preventing significant microbiome disruption; (2) rapid bactericidal activity; (3) the target is essential with highly conserved residues thus minimizing the development of resistance; (4) effective against multidrug resistant bacteria and biofilms; (5) some lysins have synergy with clinically relevant antibiotics, enhancing their potential therapeutic application (Hermoso et al., 2007). Lysins do suffer from specific limitations related to relatively short plasma half‐life, potential immunogenicity and an inability to target intracellular bacteria (Hermoso et al., 2007). Nevertheless, lysins are being evaluated clinically for their therapeutic potential.

SAL200 (Tonabacase) became the first lysin tested in humans in a phase I clinical trial. SAL200 is a recombinant derivative of the SAL‐1 lysin produced by the staphylococcal SAP‐1 bacteriophage (Jun et al., 2011). Studies have shown that SAL200 is bactericidal against encapsulated, biofilm forming and planktonic MRSA and MSSA strains (Jun et al., 2011). SAL200 retained activity in vivo when used to treat both *S. aureus* systemic infection (Jun et al., 2013) and pneumonia (Bae et al., 2019) using mouse infection models, where significant improvements in survival were observed. Satisfactory safety and tolerance profile in the phase I trial prompted further investigations including a phase IIa study for the treatment of persistent *S. aureus* bacteraemia (NCT03089697). A US‐based phase II trial has also been approved by the FDA for the treatment of complicated *S. aureus* bacteraemia and infective endocarditis (Tonabacase – iNtRon Biotechnology).

CF‐301 (Exebacase) is a clinically relevant *S. aureus* targeting lysin derived from a *Streptococcus suis* phage which exhibits broad lytic activity against MRSA, VISA and various other virulent staphylococcal and streptococcal strains (Gilmer et al., 2013). CF‐301 underwent phase II clinical trials for the treatment of complicated *S. aureus* bacteraemia (NCT03163446). Patients received CF‐301 or placebo in addition to standard antibacterial therapy. CF‐301 in combination with an antibiotic partner resulted in improved clinical outcome (70.4% vs. 60%) compared to the antibiotic only treatment group. Thirty‐day mortality rates were 9.7% for CF‐301 in combination with antibiotics and 12.8% in the antibiotic only group. Subgroup analysis showed CF‐301 in combination with antibiotics reduced the 30‐day mortality of MRSA‐infected patients by 21% (McCarthy, 2022). Recently CF‐301 was examined in a randomized, double‐blind, placebo‐controlled phase III study examining efficacy and safety in patients receiving standard antibacterial therapy for the treatment of bacteraemia and endocarditis (NCT04160468). Although the results have not been published, this trial was terminated for futility following an interim efficacy analysis.

## SMALL ACID‐SOLUBLE SPORE PROTEINS

Small acid‐soluble spore proteins (SASPs) are DNA‐binding proteins that are synthesized within hours following the onset of sporulation, and comprise a significant proportion of mature spores (Mohr et al., 1991). SASPs interact and saturate DNA in a non‐sequence‐specific manner, altering the conformation of DNA and provide protection of spore DNA from environmental stresses (Mohr et al., 1991). SASP producing bacteria encode highly conserved SASP‐specific endoproteases which are tightly regulated and only produced during spore outgrowth. An inability to degrade SASPs in nonspore formers, results in an inhibition of transcription, DNA replication and cell division culminating in cell death (Hayes & Setlow, 2001).

This has led researchers to examine the use of SASPs as alternative antibacterial agents. Rather than administrating SASPs in protein form which are unlikely to retain activity in harsh in vivo conditions, Phico therapeutics have successfully incorporated the SASP expression cassette into the genome of specific bacteriophages. The technology, referred to as SASPject, uses phage as targetable nano‐delivery vehicles where phage infection results in the precise delivery of the SASP cassette into target bacteria (Barnard & Cass, 2021). The expression of the SASP gene is controlled by a constitutive promoter originating from the target bacteria, maximizing intracellular production following injection. The *S. aureus* specific SASPject PT1.2 system maintained activity against 225 geographically diverse isolates and kill kinetic data indicated a 6log_10_ reduction within 1 h illustrating the rapid bactericidal activity of SASP‐based therapies (Cass et al., 2021). Furthermore, activity is unaffected in the presence of human serum albumin and isolates retained susceptibility following 42 days of serial passage. SASPject PT1.2 has undergone a phase I clinical trial assessing efficacy related to *S. aureus* nasal decontamination. Peer‐reviewed results of this trial have not been published, however, the company has reported that PT1.2 demonstrated a good safety and tolerability (Cass et al., 2021).

The benefits of this technology are twofold. First, since SASP interaction with DNA is sequence independent, genetic mutations are unlikely to inhibit SASP binding therefore the propensity for generating resistance to SASP activity is low. Second, since the antibacterial effect occurs through SASPs, the fate of SASPs and the rest of phage genome are independent increasing the likelihood that this system will circumvent bacterial intracellular phage defence mechanisms such as CRISPR‐Cas. This technology has the added advantage of being programmed to target specific bacteria of interest preventing significant disturbance of microbiomes.

## CONCLUDING REMARKS

*Staphylococcus aureus* has been classically considered the first ‘superbug’ owing to its combined ability to become resistant to clinically used antibiotics and express multiple virulence factors linked to disease. Despite the drastic need for new antimicrobials that target *S. aureus*, pharmaceutical innovation in this area has not been forthcoming. The last truly novel classes of antibiotics clinically to treat *S. aureus* infection were daptomycin and linezolid which were introduced in the early 2000s. Subsequent antibiotics that have been approved for use against *S. aureus* have been structural alternatives to existing well‐established antibiotic classes. While modifications to these structures have been designed to retain activity against well‐established resistance mechanisms, relying on this strategy may only delay the antibiotic resistance wave rather than overcome it.

Recent estimates put the cost of developing a new antibiotic as high as $1.5 billion (Towse et al., 2017), which has discouraged some pharmaceutical companies from investing in antibiotic development. Fortunately, government organizations, academic spin‐out companies and charitable foundations are working together to put in place new economic models to ensure future advancements and innovation. Initiatives such as the Combating Antibiotic‐Resistant Bacteria Biopharmaceutical Accelerator (CARB‐X), the Global Antibiotic Research and Development Partnership (GARDP), and the Innovative Medicines Initiative (IMI) aim to fund mid‐late hit optimization through to clinical development. A further $1 billion AMR action fund, established by the International Federation of Pharmaceutical Manufacturers and Associations, the WHO, European Investment Bank and the Wellcome Trust, aims to bring between two and four new antibiotics to the market by 2030.

**FIGURE 3:**
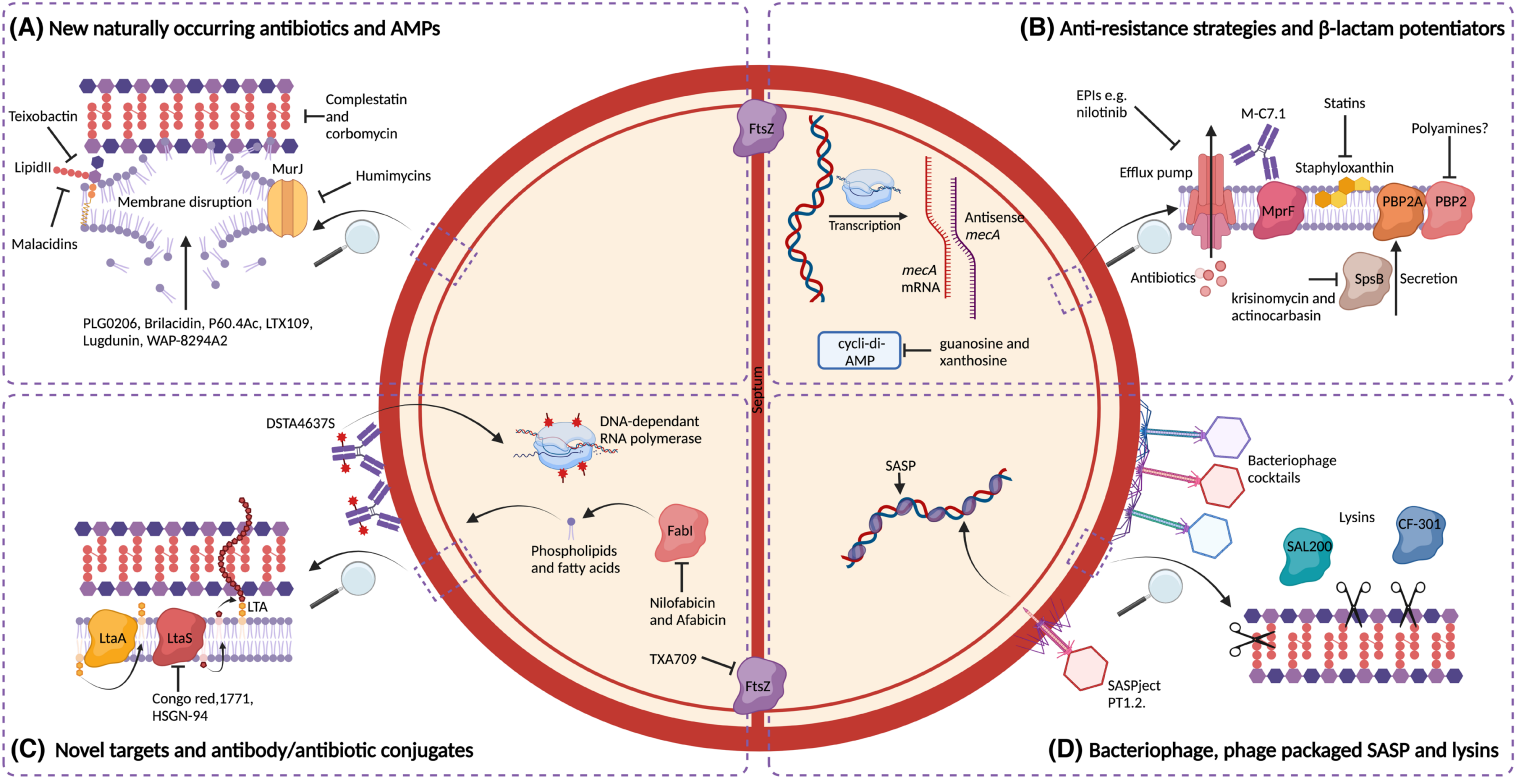
Summary of novel strategies currently being developed to treat multidrug resistant *Staphylococcus aureus* infections.

In addition to these economic incentives, biotechnological and scientific advancements related to genome mining for natural antibiotic discovery, improvements in AMP engineering and antibiotic adjuvant development, identification of β‐lactam potentiators and novel AAC construction have provided novel avenues for the development of anti‐staphylococcal therapies (Figure 3). Furthermore, the use of phage, phage lysins or phage as delivery systems for SASPs in addition to an improved understanding of fundamental staphylococcal biology directing the identification of essential proteins as drug targets (Figure 3) are fuelling innovative intervention strategies that show significant potential in combating multidrug resistant *S. aureus* infections.

## AUTHOR CONTRIBUTIONS

**Edward J.A. Douglas:** Conceptualization (equal); investigation (equal); project administration (equal); visualization (lead); writing – original draft (equal); writing – review and editing (equal). **Sri Wijayanti Wulandari:** Investigation (supporting); visualization (supporting); writing – original draft (supporting). **Scott D. Lovell:** Investigation (supporting); visualization (supporting); writing – review and editing (supporting). **Maisem Laabei:** Conceptualization (equal); funding acquisition (lead); investigation (equal); project administration (equal); supervision (lead); visualization (supporting); writing – original draft (equal); writing – review and editing (equal).

## FUNDING INFORMATION

This work was supported by the Academy of Medical Sciences Springboard Award (SBF006\1023: M. Laabei) and the Beasiswa Pendidikan Indonesia Scholarship (S.W. Wulandari).

## CONFLICT OF INTEREST STATEMENT

The authors declare no conflict of interest.
